# Methylene Blue in the Cure of Ulcerative Stomatitis

**Published:** 1909-02-27

**Authors:** 


### METHYLENE BLUE IN THE CURE OF ULCERATIVE STOMATITIS.

The local application of chemically pure methylene blue in the form of powder to the lesions of ulcerative stomatitis and tonsillitis has been strongly advocated in France, though in this country it seems to be little known. At one time it was thought to be specific for Vincent's angina, but there is evidence to show that it may bring about very rapid improvement and cure in other ulcerative affections of the bucco-pharyngeal mucosa also. Chauffard, Siredey, and Simonin have recorded instances to this effect. Even in very severe cases the benefits to be obtained from the local application of pure methylene blue in powder form are sometimes quite remarkable. Two instances will illustrate this.

A young woman, 24 years of age, who had for ten years or more been subject to sore throats but had never been so severely affected before, began to complain of her throat being painful, especially on swallowing, on December 8. A week later the act of deglutition had become easier, but there was mild pyrexia in the evening, accompanied by anorexia, a tendency to shiver, and at the same time to perspire. Notwithstanding the use of antiseptic gargles the soreness of the throat increased, and deglutition became more and more difficult, the breath and the expectoration became fœtid, and occasionally the sputum was tinged with blood. On December 24 the condition was as follows: There were many enlarged and painful glands both in front of and behind the angle of the right lower jaw. Breath and sputum were fœtid to such a degree as to be noticeable by the patient herself. Upon the antero-internal portion of the right tonsil there was greyish ulceration with sanious discharge, rather resembling a chancre. The ulcer looked as if it were penetrating deeply into the tonsil. It was rather less in area than is a threepenny piece. Except for an acutely reddened zone around this ulcer there was nothing else abnormal about the throat. The uvula, the other tonsil, and the gingivo-buccal mucosa were healthy. The urine contained no albumen.

Bacteriological examinations were at once made, and the case was shown to be a clear instance of Vincent's angina. The two micro-organisms whose symbiosis seems essential to the development of this disease were present in almost pure culture—typical spirilla and long fusiform bacilli, staining with carbol methylene blue but not by Gram's method. No Klebs Lœffler bacilli were obtained.

Treatment by chemically pure methylene blue applied in powder form to the ulcerated surface was started forthwith, and the effects were almost instantaneous. The first application was on the 24th without subsequent lavage. The next morning the appearance of the throat had entirely changed. The centre of the ulcer was still greyish, but buttons of granulation tissue were already showing. The edges had become less angry looking, and there was no doubt as to repair having begun. The dysphagia was less and the patient felt easier three or four hours after the application. The second dose was given on the 25th, followed six or seven hours later by a lavage with boiled water. On the 26th there was no longer any pyrexia, and the patient could swallow with ease. The submaxillary lymphatic glands were still swollen, but less so than before, and they were no longer painful. The tonsillar ulceration was now represented merely by a depression, for all the grey slough and the sanious discharge had disappeared. Neither breath nor sputum was fœtid any longer.

Two applications of the methylene blue had clinically produced a cure. Bacteriologically the result was similar, for spirilla and fusiform bacilli had become quite scarce, whereas before this they were present in myriads. To make quite sure a third application was made upon the 26th, and this completed the cure. It may be added that the methylene blue was applied generously and as deeply as possible into the base of the ulcer. It was very well borne, the only particular point to note being that it produced a moderate burning sensation that lasted for about five minutes each time.

Another case was as follows: A young man was brought to the hospital suffering from the most violent ulcero-membranous stomatitis. The gums and the inner aspect of the cheeks were the seats of extensive ulcerations which were covered by thick false membranes.

Bacteriological examinations of the membranes revealed the presence of streptococci and various other micro-organisms, but neither diphtheria bacilli, spirilla, nor bacilli fusiformes. Owing to absence of the latter methylene blue was not at first tried; cauterisations with chromic acid in the form of crystals were ordered. The acid was applied with all the usual precautions, but the patient, who was extremely nervous, refused its repetition, and left the hospital in fear of its being used again. He was seen next day, the stomatitis being quite as severe as ever.

Chemically pure methylene blue was resorted to as a local application over the whole of the ulcero-membranous areas. The same evening there was an undoubted reduction in the amount of false membrane, and the ulcers were looking much less angry than before. The applications were repeated morning and evening, and at the end of three days the patient was well and remained cured.

